# Towards a Deeper Understanding of Creep and Physical Aging Behavior of the Emulsion Polymer Isocyanate

**DOI:** 10.3390/polym12061425

**Published:** 2020-06-26

**Authors:** Shihao Zhou, Xuansheng Fang, Yaolong He, Hongjiu Hu

**Affiliations:** 1 Shanghai Institute of Applied Mathematics and Mechanics, School of Mechanics and Engineering Science, Shanghai University, Shanghai 200072, China; zhoushihao9370@foxmail.com (S.Z.); friendship2000@163.com (X.F.); yaolonghe@shu.edu.cn (Y.H.); 2 Shanghai Key Laboratory of Mechanics in Energy Engineering, Shanghai 200072, China

**Keywords:** emulsion polymer isocyanate, creep, physical aging, free volume, positron annihilation lifetime spectroscopy

## Abstract

Information of the relaxation behaviors of polymer film is crucial to judge the durability of emulsion polymer isocyanate (EPI) as a structural adhesive for bonding timber-based products. A sequence of tensile creep tests and free volume evaluation of the cured EPI adhesive films during isothermal condition were carried out by dynamic mechanical analysis and positron annihilation lifetime spectroscopy, respectively. It is the first time to explore the creep response and physical aging of the EPI film, as well as associated microstructural evolution. The results indicate that the creep characteristics of the glassy EPI coating intimately depend upon the crosslinker and elapsed time, and the ideal momentary creep master curve can be constructed in terms of modified horizontal shifting method. Furthermore, the relaxation process is found to be dominated by vacancy diffusion mechanism. In addition, increasing the polymeric isocyanate content can significantly enhance the resistance to creep deformation of EPI films, but also accelerate the physical aging process. Due to a higher packing degree of pure polymer films, the EPI films with aqueous emulsified isocyanate exhibit much better relaxation resistance compared to that with general isocyanate crosslinker.

## 1. Introduction

As a result of excellent mechanical properties, heat resistant, water resistant, and being environmentally-friendly, emulsion polymer isocyanates (EPI), which are regarded as a novel structural adhesive, are widely used in the production of modern wood products, such as EPI-bonded structural-composite lumber and cross-laminated timber over the past several decades [1,2]. For the purpose of maintaining the structural integrity of bond-line joining wooden adherends, great efforts have been made to enhance the bonding performance and water resistance of EPI adhesive [2,3,4,5,6,7,8]. However, there is only a handful of scientific and industrial interests in discussion of the mechanical properties of the cured polymer films. Taki et al. carried out the pioneering investigation on EPI film [9,10]. They studied the effect of cross-linking densities on the mechanical behavior of adhesives films over a wide temperature range. By means of dynamic mechanical analysis (DMA), Umemura et al. also reported the effect of temperature on the storage modulus of cured EPI films [11]. We investigated the influences of the type and loading of crosslinkers, as well as curing process on the quasi-static and dynamic mechanical responses of EPI film in humid environment [12]. Ling et al. found that post curing could reduce the residual isocyanate group (–NCO), and thus give rise to pronounced changes in the viscoelastic behavior of EPI films [13]. Recently, we studied the sodium dodecyl sulfate dependence of mechanical performance of polyvinyl-acetate-based EPI [14]. As a protective colloid and the main provider of hydroxyl groups, the poly (vinyl alcohol) (PVOH) is an essential component in EPI. Due to the fact that the glass transition temperature of PVOH is greater than 70 °C, an EPI cured film naturally exhibits the glassy state at room temperature. Notwithstanding, with the addition of isocyanate crosslinker, the molecular structure of the EPI adhesive layer experiences a certain degree of spatial network distribution, it may reveal a creep characteristic. In terms of in-situ nano-indentation, Rindler et al. observed that the cured EPI revealed more obvious creep and relaxation in respect to urea formaldehyde and formaldehyde amino adhesives [15]. Moreover, compared with the zero to marginally creeping phenolic resorcinol-formaldehyde adhesives, the published work reported that the strain response of the EPI glue line would considerably evolve against time under an external force, leading to the premature failure of structural timber bonding [16]. Thus, it is crucial to further understand the underlying viscoelastic nature of EPI to find the conditions that affect the relaxation of cured film and hence to suggest mitigation methods to boost the life of the water-based polymer adhesive. However, very few studies have thoroughly explored the influences of crosslinkers and storage time on the creep behaviors of EPI film.

As the thermoplastics have experienced a cooling from melting temperature during the solidification run, the cured glassy polymers are usually in a nonequilibrium state. The material system with excess thermodynamic quantities would spontaneously evolve towards the equilibrium referred to as the physical aging [17], which would lead to the variation in microstructure and macroscopic properties under storage and usage conditions. Therefore, intensive attentions have been paid to investigate the time-dependent properties of aging glassy, and excavate the potential relaxation mechanism of the 1970s [17]. It is well recognized that physical aging is accompanied not only by the increase in density, dielectric constant, refraction index, stiffness, yield stress, and viscosity, but also by decrease in enthalpy, ductility, ultimate elongation, permeability, and also absorption capacity of the polymer and its composites. The aging rate was found to be dependent upon many factors, including environmental temperature and relative humidity [18], as well as the film thickness [19], doped nanofiller [20,21], and molecular weight [22] of polymer, even experienced CO_2_ exposure [23,24], stress history [25], etc. The details can be seen in the excellent reviews by Hutchinson [26] and Cangialosi et al. [27]. Due to the rapid evaporation of solvent (water), and the chemical crosslinking reaction between the hydroxyl groups (–OH) in the polymer and isocyanate groups (–NCO) in the preparation process [28], the amorphous glassy cured EPI film may pass through a nonequilibrium state, in which the free volume and microstructure would vary over time. That is to say, the physical aging may occur in the EPI bonding layer. The material becomes stiffer and more brittle because of a naturally-occurring densification of the structure, resulting in weakening the resistance to rupture of EPI bonded wood products. Therefore, understanding the relaxation behavior of cured EPI films is beneficial for quantitatively assessing the long-term durability and dimensional stability of the adhesively bonded joint, and also is the basis for the proper engineering design and development of high-performance adhesive. Unfortunately, there is no previous literature discussing the physical aging of EPI film.

In consequence, this present study performed a series of tensile creep experiments and positron annihilation lifetime spectroscopies (PALSs) for the cured EPI films, and the effects of type and loading of isocyanate crosslinker on the polymer viscoelasticity were discussed in detail. In terms of the evolution of momentary creep compliance with various storage time, the physical aging of EPI adhesive was unveiled for the first time, and the corresponding aging mechanism was further explored according to the elapsed time dependence of free volume fraction. In addition, based on the aging time related shape factor of Kohlrausch–Williams–Watts (KWW) equation, we developed a modified horizontal shift method to predict the mechanical response of EPI cohesive with different crosslinker throughout long-term service.

## 2. Experimental

### 2.1. Materials and Samples

(1) Aqueous polymer emulsion (EPI main component): a vinyl acetate based tri-copolymer latex (xPVAc, EP203), containing approximately 30% (wt.) of CaCO_3_, as well as 10% (wt.) of PVOH as a protective colloid and the main provider of hydroxyl groups. The aqueous vinyl latex was prepared by Shanghai Junyan Chemical Material Co. Ltd. (Shanghai, China) and was used as received. It had a solid content of 51.0%, viscosity of 7100 mPa·s (at 25 °C), and pH of 6.5.
(2) Polymer isocyanate (EPI crosslinkers): EMDI (Rubinate 9259) and p-MDI (Rubinate 5005), of which characteristics as listed in Table 1, were synthesized by Huntsman Polyurethanes (China) Ltd. and also were employed as received.

The water-based polymer emulsion were mixed by an electric agitator with the isocyanate crosslinker at the mass ratio of 100:0, 100:5, 100:10, 100:15, and 100:20, respectively. After degassed for 30 s, the adhesive were spread on Teflon-coated glass plate and placed in a dried chamber at 35 °C for 72 h. Followed by, the obtained films were post-cured at 140 °C for 24 h to eliminate the residue of isocyanate groups (–NCO). Finally, all the samples with dimensions of 20 mm × 5 mm × 0.1 mm, were preserved in a desiccator with recently dried silica gel at room temperature.

### 2.2. Test Methods

#### 2.2.1. Uniaxial Tensile Creep

**Figure 1:**
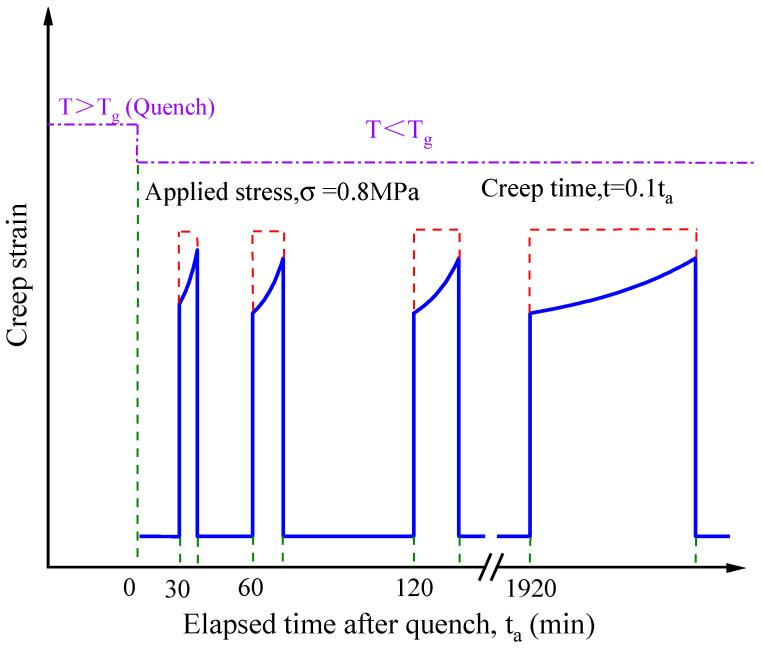
Sequence of tensile creep tests.

The mechanical measurements were carried out at the temperature of 30 °C, humidity of 5% RH by a film/fiber tension clamp on a DMA (TA Q800) with a DMA-RH Accessory. Firstly, EPI films were heated to the temperature $T_{0}=100$ °C in the environmental chamber of DMA and kept for 30 min to remove the heat history, and then rapidly cooled to 30 °C within 5 min, and maintained at that temperature for different storage times ($t_{a}$). Subsequently, the tensile creep tests of cured EPI films were implemented following the snapshot assumption suggested by Struik [17] as indicated in Figure 1 at the elapsed time of 30, 60, 120, 240, 480, 960, and 1920 min. In addition, the loading procedure was at a stress of 0.8 MPa in order to the linear viscoelastic requirement, and each creep time (t) is the previous aging time of 10%. That is to say, the recovery time is long enough.

#### 2.2.2. PALS Measurements

Positron annihilation lifetime spectroscopies (PALSs) were carried out at the ambient temperature on all the above-stated EPI samples, by exposing them to radioactive ^22^NaCl enveloped between 25-μm Kapton foil. The source was sandwiched between 10 layers of the membrane with a total thickness of 1 mm on each side to ensure that most of the positrons completely annihilated inside the EPI film. A fast-fast coincidence circuit of the PALS spectrometer at the IRI-TU Delft, was used to record all PALS spectra. Each spectrum was collected to a fixed total count of 2 × 10^6^, which is high enough to get a good analysis but at the same time low enough to avoid any substantial radiation effects. The spectrums were analyzed using the program PATFIT [29], and were resolved into three lifetime components: a short one of 125 ps related to para-positronium (p-Ps) annihilation, an intermediate one of 400 ps related to free positron annihilation, and a long one component related to ortho-positronium (o-Ps) annihilation. In order to inhibit the potential radiation effect, which may decrease the intensity ($I_{3}$) of o-Ps due to the electric field built-up [30,31], the positron source was grounded in the following PALS measurements. As the lifetime spectra is collected in 2 h time intervals, the elapsed time before PALS test is set to be larger than 20 h in order to meet Struik’s snapshot assumption.

## 3. Results and Discussion

### 3.1. Crosslinker Dependence of Creep Characteristics

**Figure 2:**
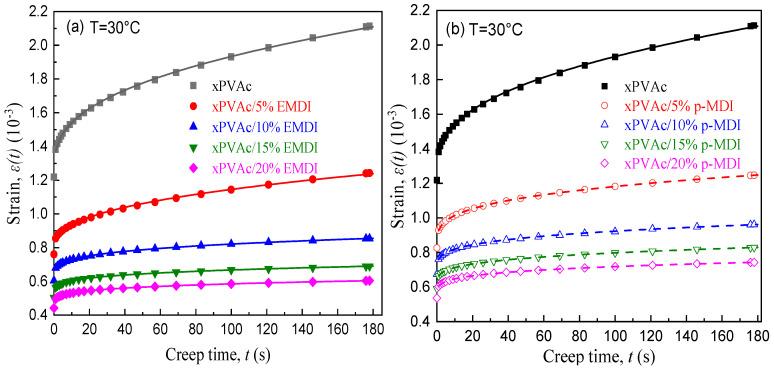
Tensile creep curves of cured EPI films with various isocyanate crosslinkers at $t_{a}=30$ min (Dots: experimental data; Curves: fitted by the Kohlrausch–Williams–Watts (KWW) equation).

The structural stability of EPI bonded products subjected to constant load for long periods of time is mainly controlled by resistance of the adhesive layer to creep. Hence, a series of uniaxial tension experiments under a constant stress of 0.8 MPa were accomplished on the EPI cured film, which were crosslinked by polyisocnanate with different concentration (0%, 5%, 10%, 15%, and 20%). The obtained tensile strain ε(t) versus loading time t curves of EPI samples as seen in Figure 2a,b. It is clear to see that all of the films exhibit the typical creep response. As the creep time t increases, the values of ε(t) rapidly go up at the initial stage, following that, the increase extent of the tensile deformation gradually decreases. Due to the thermoplastic polymer characteristic, the cured films of xPVAc yield significant creep strain compared to the EPI. Adding a small amount of isocyanate crosslinker (5%) would greatly boost the creep resistance by about 160%. Such a result is in agreement with the difference in creep data between EPI and PVAc reported by Rindler et al. based on the nanoindentation [12]. As might be expected, the crosslinkers have the significant effect on the ε(t)−t curves. With the increase of polyisocyanate content from 5% to 20%, the creep strain of xPVAc/EMDI film steeply reduces by a factor of nearly 2. The trends of crosslinker-dependent creep results for xPVAc/p-MDI samples are similar to EPI films with EMDI but relatively less improvement, indicating that EMDI seems to have the better crosslinking capacity.

In terms of our previous work [12], the present creep behaviors of cured EPI films are in the range of linear viscoelasticity, where the creep compliance $J\left(t\right)=\varepsilon \left(t\right)/\sigma_{0}$ would not rely on the stress level. Hence, the evolution of J(t) is attempted to analyze by Kohlrausch–Williams–Watts (KWW) equation as follows:

(1) $$J\left(t\right)=J_{0}\exp{\left(\frac{t}{\tau }\right)}^{\eta }$$

where $J_{0}$ is the initial creep compliance (t=0), *t* is the creep time, τ is the characteristic retardation time, and η is the shape factor for the creep curves.

Above, Equation (1) is used to fit all the creep test curves of EPI film crosslinked by EMDI or p-MDI. The simulations by 1stOpt^®^ non-liner regression software are also demonstrated in Figure 2, where the fitted data (lines) are in good agreement with the experimental results (dots). The regression related coefficients very close to 1 means that the KWW model is feasible for capturing the strain response of cured EPI films during the creep process. Thus, one can further elaborate the dependence of aforementioned creep behavior on the crosslinker based on the KWW parameters ($J_{0}$, τ, and η) as following sections.

**Figure 3:**
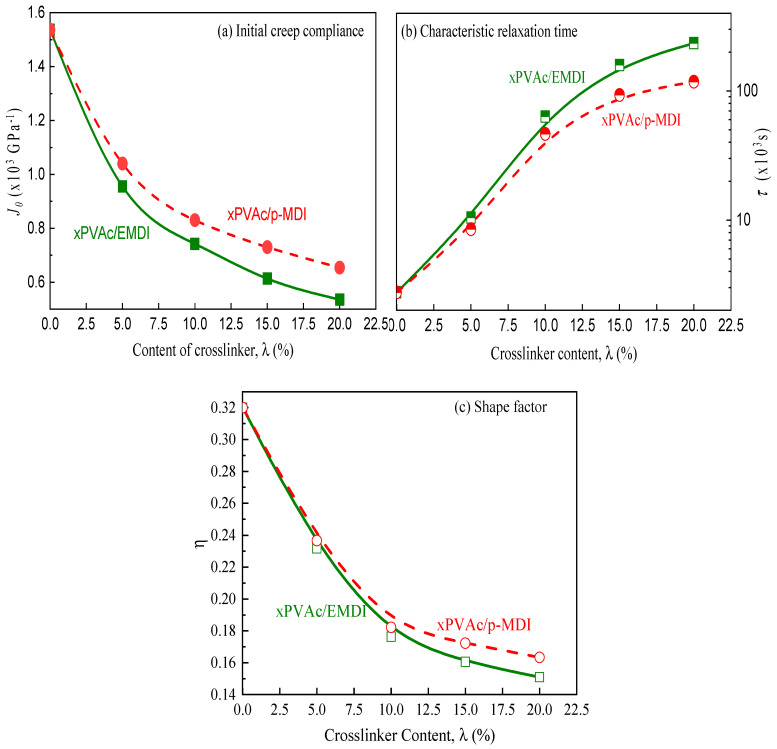
Effect of isocyanate crosslinkers on the KWW parameters.

Based on aforesaid tensile creep curves of cured film and Equation (1), the influences of isocyanate crosslinkers on the initial creep compliances $J_{0}$, characteristic retardation time τ, and shape factor η for EPI films are obtained as shown in Figure 3a–c, respectively. It is found that with an increase of the isocyanate content in the EPI system, the crosslinking density of cured film could be enhanced and the molecular network rigidity accordingly enlarges, while the free motion of the polymer segment is constrained, contributing to a steep decrease in the magnitude of $J_{0}$, and a significant growth in the τ value. Compared to the uncrosslinked main component films, the average characteristic relaxation time for xPVAc/20%EMDI and xPVAc/20%p-MDI rise by factors of up to about 85 and 43, respectively, because the cured films are substantially changed from a thermoplastic polymer to thermoset resin. In addition, the raised crosslinker concentration causes the η for the cured materials to dramatically decrease. That is to say, the relaxation spectrum broadens progressively, which may be ascribed to the complexity of chemical reaction between the high active isocyanate (–NCO) and the aqueous polymer emulsion that has a variety of functional groups such as hydroxyl, carboxymethyl, amide, amine, etc. Furthermore, in respect to the standard p-MDI, the aqueous emulsifiable isocyanate has stronger disperse ability and cross-linking effects, resulting that the EPI films with EMDI exhibit relatively less $J_{0}$ and η, but the longer relaxation process. It can be further verified by the obvious difference of micro-structure in the EMDI crosslinked films and the film with p-MDI.

In order to promote understanding of the creep response for cured EPI films, we took the first derivative of Equation (1) with respect to the creep time as follows.

(2) $$\overset{\cdot }{J}\left(t\right)=\frac{J_{0}\eta }{\tau }\cdot \exp{\left(\frac{t}{\tau }\right)}^{\eta }\cdot {\left(\frac{t}{\tau }\right)}^{\eta -1}$$

And taking the logarithm of both sides of Equation (2) yields the following Equation (3).

(3) $$\begin{array}{l} \ln\overset{\cdot }{J}\left(t\right)={\overset{\cdot }{J}}_{c}+\left(\eta -1\right)\ln t+{\left(\frac{t}{\tau }\right)}^{\eta }\\ {\overset{\cdot }{J}}_{c}=\ln\frac{J_{0}\eta }{\tau^{\eta }} \end{array}$$

**Figure 4:**
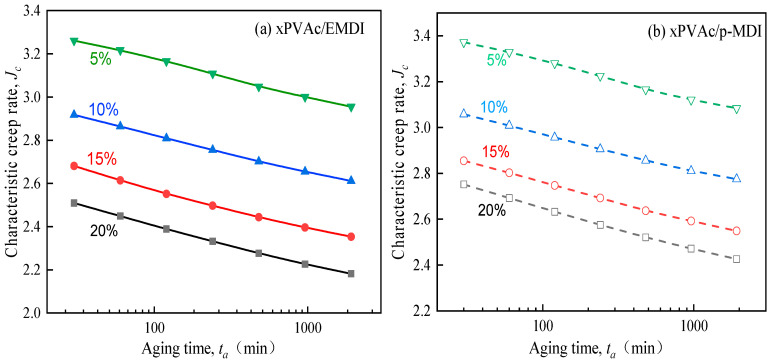
Effect of isocyanate crosslinkers on the characteristic creep rate for cured EPI films.

Noticeably, ${\overset{\cdot }{J}}_{c}$ which is independent of the creep time, and only relates to the three KWW parameters, which are dependent of the share and type of the isocyanate as seen Figure 4a,b. It is observed that the characteristic momentary creep rate sharply decreases with increasing aging time, indicating that the aging process pronouncedly restrains the creep behavior of EPI film. Meanwhile, adding isocyanate crosslinker would also reduce the value of ${\overset{\cdot }{J}}_{c}$, and the higher the loading of p-MDI or EMDI, the slower the creep process of glassy EPI films. This great difference may result from different crosslink density and the glass transition of EPI films, which curing effect significantly improves as more isocyanate is added as referred to in our previous work [12].

### 3.2. Effect of Physical Aging on Creep Behaviour

As the water-based polymer emulsion is typically added by a certain amount of polyvinyl alcohol (PVOH), the cured EPI film may usually exhibit the glassy state at the room temperature. The thermodynamic nonequilibrium induced by the adhesive solidification would result in the variation in amorphous polymer microtopography. The acquisition of creep behavior during aging process and the related quantitative analysis should be vital tasks of EPI research.

**Figure 5:**
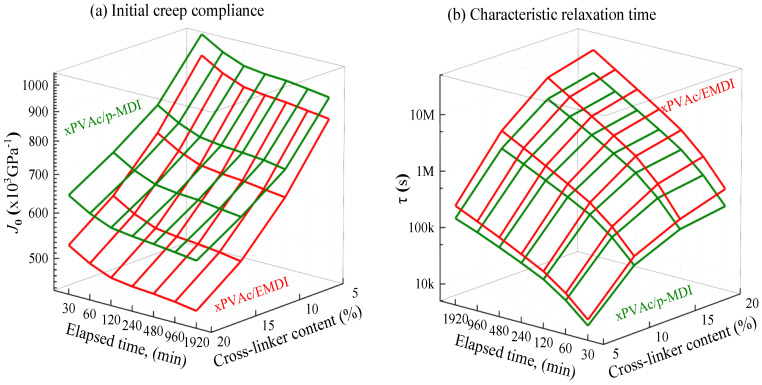

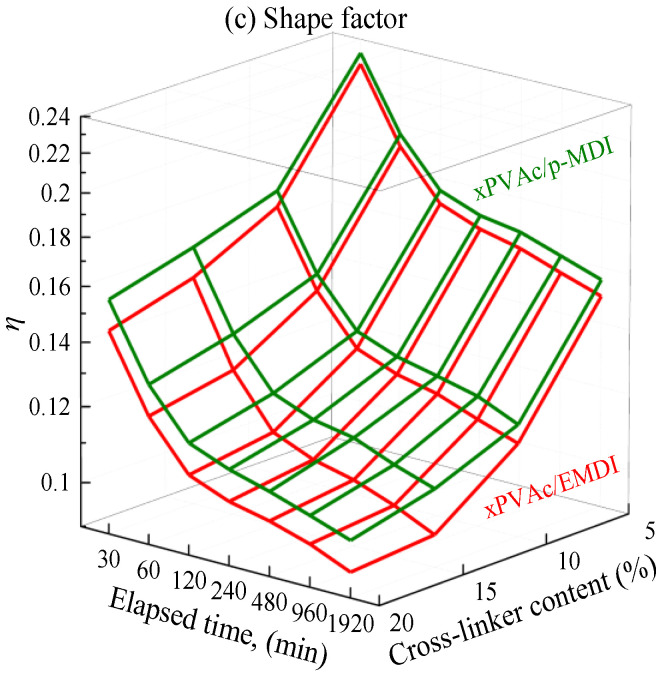
Effect of aging time on the KWW parameters for EPI with different crosslinkers.

It is expected that the momentary creep curves of EPI film during isothermal physical aging can be fitted very well by KWW formula, and the evolution of creep characteristics with various elapsed time ($t_{a}$), are shown in Figure 5a–c. As observed from the KWW parameters curves for all of the samples, with increased $t_{a}$, the magnitudes of $J_{0}$ markedly decrease, whereas the τ values steeply ascend, indicating a significant physical aging occurs in cured aqueous adhesive films, even for the EPI with isocyanate crosslinker content of up to 20%. In other words, physical aging can give rise to the significant change in creep behavior of glassy EPI film.

**Figure 6:**
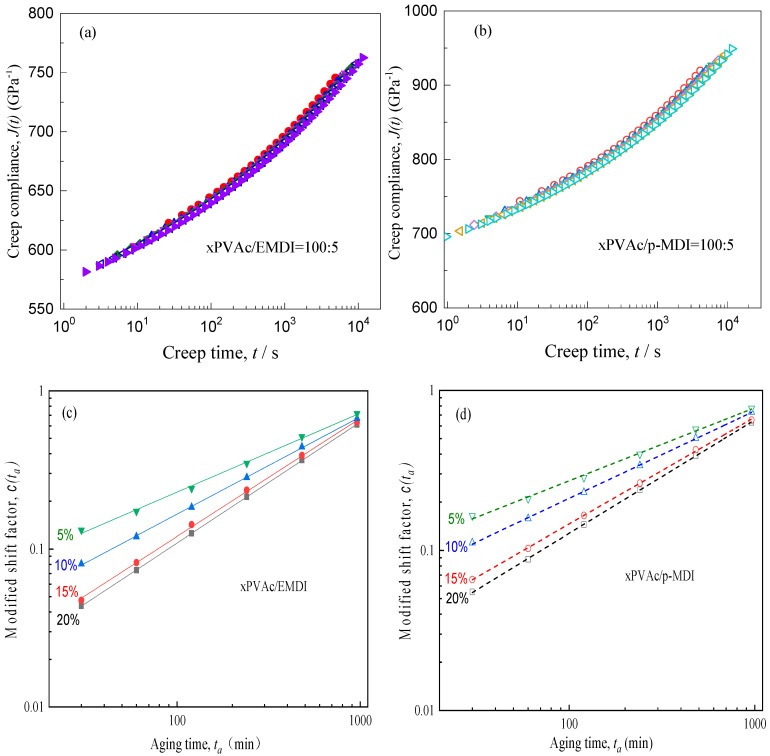
Creep master curve and shift factor of EPI films with different crosslinkers.

Note that the shape factor of creep curve would decrease as the amount of isocyanate increases. Meanwhile, for the cured film at a given type and content of crosslinker, η gradually diminishes in the process of physical aging. It may ascribe that physical aging would lead to a dramatic drop in the fractional free volume inside the amorphous region of EPI film. A major consequence is that the internal packing degree enlarges and the molecular chain realignment would gradually tighten, while the mobilities of the chain segment is weakened and the stiffness of EPI film increases, resulting to an increased relaxation spectrum of glassy. Furthermore, it suggests that all curves of J(t) against time for aging EPI have different η and cannot be superimposed by Struik’s horizontal shifts [17]. Based on the effective time theory [17], a modified horizontal shift factor has been proposed as shown in Equation (4) to superimpose these creep curves of aged EPI. For the convenience of processing data, the creep curve at the longest aging time (1920 min) is selected as the reference, the compliance data at other aging times are shifted along the log-time axis by Equation (4) to construct the creep master curves. The shifting results and associated shift factor $c\left(t_{a}\right)$ are shown in Figure 6a–d.

(4) $$c\left(t_{a}\right)=\frac{t_{a}}{t_{ref}}={\left\{\frac{\tau \left(t_{ref}\right)}{\tau \left(t_{a}\right)}\times 10^{\left[\frac{\eta \left(t_{a}\right)-\eta \left(t_{ref}\right)}{\eta \left(t_{ref}\right)}\right]}\right\}}^{-1}$$

where $c\left(t_{a}\right)$ is the modified horizontal shift factor and $\tau \left(t_{ref}\right)$ and $\eta \left(t_{ref}\right)$ are the characteristic retardation time and shape factor for EPI at the reference aging time, $t_{ref}$.

The smooth master curves of EPI at given type and 5% content of crosslinker as seen Figure 6a,b, and the EPI films with other loadings of isocyanate also reveal the similar trends, indicating the validity of the time-aging time superposition principle. Moreover, it is can be further verified from Figure 6c,d, wherein the evolution of $c\left(t_{a}\right)$ value versus aging time in double logarithmic coordinate, all point lie on a straight line with different slope ($\mu =dlogc\left(t_{a}\right)/dlogt_{a}$) for xPVAc crosslinked by EMDI and p-MDI, respectively. Notably, the magnitude of μ increases with an increase in isocyanate loading for the two kinds of EPI films, indicating that the addition of crosslinker has accelerated aging process for xPVAc. This abnormal phenomena may be ascribed to the fact that a great deal of NCO groups have reacted with water molecule in the polymer emulsion during solidification, leading to the presence of many mesoporous in the adhesive coating layer due to the carbon dioxide (CO_2_) gas arising from this reaction, as shown in Equation (5) [12]. As a result, the generated nano-scale cavities may greatly increase the formation and annihilation of positronium in EPI polymers. Due to the better dispersion in the emulsion and the higher reaction with water molecules, aqueous emulsified isocyanate may produce more distributed micropores in cured EPI film than in contrast to standard industrial p-MDI. Therefore, xPVAc/EMDI film exhibit higher aging shift rate compared to xPVAc/p-MDI at the constant addition of crosslinker.
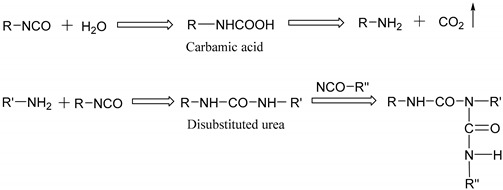
(5)

### 3.3. Relaxation Mechanism during Physical Aging

As previously mentioned, the crosslinker and physical aging play a significant role on the macroscopic mechanical performance of EPI coating. However, the potential effect mechanism is still very unclear. Accordingly, PLAS test was carried out for both xPVAc/EMDI and xPVAc/p-MDI, respectively, with the aim of elucidating the following essential questions: (i) How do isocyanate and aging affect the free volume cavities? (ii) Which mechanism controls the relaxation process, and how to quantitively describe the evolution of free volume in cured EPI film during physical aging?

**Figure 7:**
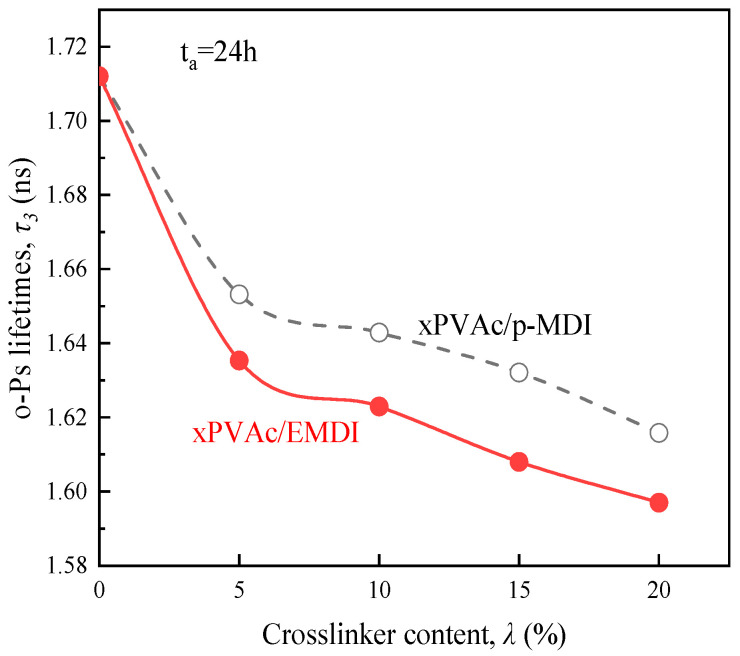
Effect of isocyanate crosslinkers on the lifetimes *τ*_3_ of o-Ps in aging EPI film.

In the light of the measured positron lifetime spectra, the o-Ps lifetimes ($\tau_{3}$) for the aging adhesive films are plotted in Figure 7. Evidently, the higher the isocyanate loading, the shorter the pick-off lifetime of o-Ps for EPI film. Compared to xPVAc/p-MDI film, the $\tau_{3}$ values for xPVAc/EMDI are relatively lower at the same crosslinker content. It suggests that the EPI film crosslinked by EMDI contains the smaller radius of the cavities in polymer than that crosslinked by p-MDI.

**Figure 8:**
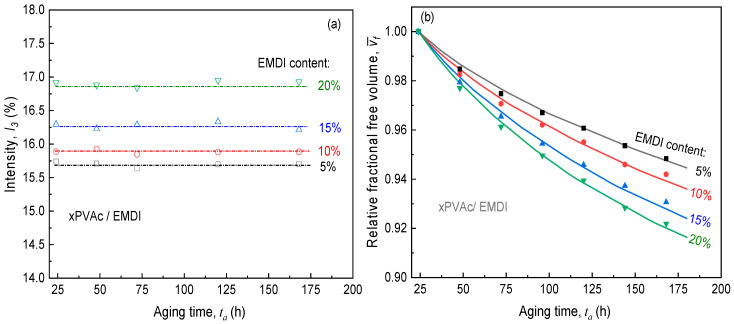
Evolution of (**a**) intensity *I_3_* of o-Ps and (**b**) relative fractional free volume (Dots: experimental data; Curves: calculated by vacancy diffusion model) in cured EPI film during physical aging.

Before discussing the evolution of free volume in EPI film during the physical aging, we check the intensity ($I_{3}$) of o-Ps against different aging time ($t_{a}$) as shown in Figure 8a. For each xPVAc/EMDI films with given isocyanate crosslinker loading, the $I_{3}$ values keep a constant as $t_{a}$ increases from 24 to 168 h, which means that grounding the source suggested by Li and Boyce [30] could effectively minimize the accumulation of charges in the polymer during the measurement. Therefore, the PALS test may capture the variation of the size and concentration of free volume cavities in EPI films. It is widely recognized that fractional free volume ($v_{f}$) can be determined by the following equation.

(6) $$v_{f}=ƛI_{3}\frac{4}{3}\pi R^{3}$$

where ƛ is the material constant, and R is the cavity radius, which can be determined by the following equation.

(7) $$\tau_{3}=\frac{1}{2\left[1-\frac{R}{R+1.656}+\frac{1}{2\pi }\sin\left(\frac{2\pi R}{R+1.656}\right)\right]}$$

According to Equation (7), we can obtain the fractional free volume ($v_{f}$) in the water-based polymer with different isocyanate contents. Taking the value of $v_{f}$ in EPI film aged at 24 h as a reference, the evolution of relative fractional free volume ($\bar{v_{f}}$) is indicated in Figure 8b. It is found that increasing the content of EMDI would boost the free volume, implying that addition isocyanate crosslinker would promote the physical aging process for xPVAc. However, EPI film still exhibits the better resistance to creep response owing to the chemical cross-link abilities of –NCO group with hydroxyl, carboxymethyl, amide, amine, etc. The variation of $\bar{v_{f}}$ for cured xPVAc/EMDI films in the process of physical aging can also be seen in Figure 8b. For all the film, the magnitudes of $\bar{v_{f}}$ reduce over aging time as would be expected. Moreover, the attenuation rate is initially the highest and then gradually falls on account of a pronouncedly self-delaying process for the thermodynamic quantities such as entropy and enthalpy in the progress of physical aging. It is noticed that all the EPI films revealed the similar manner of the steep decay in the free volume as the aging time passes. Furthermore, a raised loading of isocyanate causes the enlargement in the value of $dv_{f}/dt_{a}$, which is clearly consistent with the evolution of the modified horizontal shift factor of EPI film with isocyanate content as seen in Figure 6.

On the basis of free volume concept, extensive theoretical and experimental efforts have been dedicated to disclosing the aging mechanism of glassy polymers [31,32,33,34,35]. Up until now, the decaying of free volume related to physical aging aggregates into three mechanisms: (i) vacancy diffusion model, (ii) lattice contraction model, and (iii) coupled lattice contraction and vacancy diffusion. Although there is still much debate over whether physical aging depends primarily on the vacancy diffusion mechanism or a combination of mechanisms, we attempted to adopt the vacancy diffusion model to describe the aging-related free volume due to its easily solved mathematic expression as seen in Equation (8).

(8) $$\frac{\partial v_{f}}{\partial t_{a}}=\frac{\partial }{\partial x}\left(Ae^{-B_{f}/v_{f}}\frac{\partial v_{f}}{\partial x}\right)$$

(9) $$v_{f}=v_{f_{i}},\;\mathrm{at}\;t_{a}=0\;\mathrm{for}-l/2<x<l/2$$

(10) $$v_{f}(\pm l/2,t_{a})=v_{f_{e}}$$

where A and $B_{f}$ are the material parameter, and the initial conditions as well as boundary conditions is determined by Equations (9) and (10), respectively.

The free volume in EPI film obtained by PALS exhibits a nonlinear decay in the process of physical aging, where the driving force is the free volume gradient. Such a decrease can be described by the second equation of Fick, where the diffusion coefficient of free volume holes is expressed by Doolittle equation ($D=Ae^{-B_{f}/v_{f}}$) [36]. The calculation results show that relative fractional free volume in xPVAc/EMDI films rapidly decreases as the aging time increases. Meanwhile, the more isocyanate that is added, the higher the aging rate of the cured film. That is, compared to the experimental data, Equation (8) adequately captures the shape and magnitude of the aging tendency as shown in the curves for EPI film in Figure 8b. In other words, the self-retarding contraction may be neglected for aged EPI film with the thickness of 100 um. Consequently, one may deduce that the free volume diffusion to the external surface dominates the physical aging process of cured EPI films.

## 4. Conclusions

(1) The addition of isocyanate crosslinker can significantly enhance the creep resistance of the waterborne polymer xPVAc, and the positive effect can boost with increasing isocyanate content.
(2) All of the cured EPI film with different crosslinkers exhibit the pronounced physical aging phenomena. However, the relaxation rate would be greatly accelerated by the introduction of the higher amount of EMD or p-MDI.
(3) Physical aging contributed to the decrease in elastic creep compliance and shape factor, while an increase in retardation time. Accordingly, the ideal momentary creep master curve can be obtained using modified horizontal shift for aging EPI film.
(4) The size and number of free volume cavities in cured EPI adhesive layers are highly dependent upon the crosslinker concentration and elapsed time, and the mechanism of vacancy diffusion controls the relaxation behavior of glassy EPI films.
(5) As aqueous emulsified isocyanate has the stronger crosslinking capacity with the functional groups available in/on the main component than the unmodified polymeric isocyanate, xPVAc/EMDI films are much better resistant to creep performance compared to xPVAc/p-MDI at the same crosslinker loading. However, it exhibits the worse resistance of physical aging, which may be credited to the higher fractional free volume in the EPI film.

## Figures and Tables

**Table 1:** Characteristics of polymer isocyanate (emulsion polymer isocyanate (EPI) crosslinkers) [12].

Crosslinker	Solid Content (%)	NCO (%)	Functionality	Modification Method
Rubinate 9259	100	30.6	2.7	Aqueous emulsifiable
Rubinate 5005	100	30.5–32.5	2.6–2.7	Standard industrial

## References

[1] Grøstad K., Pedersen A. (2010). Emulsion Polymer Isocyanates as Wood Adhesive: A Review. J. Adhes. Sci. Technol..

[2] Hu H., Liu H., Zhao J., Li J. (2006). Investigation of Adhesion Performance of Aqueous Polymer Latex Modified by Polymeric Methylene Diisocyanate. J. Adhes..

[3] Sakurada S., Miyazaki H., Hattori T., Shiraishi M., Inoue T. (1976). Adhesive Composition Consisting of Polyvinylalcohol Solution or Polyvinylacetate Latex Modified with Hydrophobic Solution of Isocyanate Compound. U.S. Patent.

[4] Qiao L., Easteal A.J., Bolt C.J., Coveny P.K., Franich R.A. (2000). Thermomechanical analysis and performance tests of some EPI wood adhesives. Pigment Resin Technol..

[5] Hu H., Zhu W., Liu H., Zhao J. (2006). Investigation on fracture performance of aqueous polymer isocyanates in terms of energy release rate. J. Adhes. Sci. Technol..

[6] Gao Z., Wang W., Zhao Z., Guo M. (2010). Novel whey protein-based aqueous polymer-isocyanate adhesive for glulam. J. Appl. Polym. Sci..

[7] Krystofiak T., Proszyk S., Jozwiak M. (2003). Studies of some properties of EPI adhesives. For. Wood Technol..

[8] Bastani A., Adamopoulos S., Koddenberg T., Militz H. (2016). Study of adhesive bondlines in modified wood with fluorescence microscopy and X-ray micro-computed tomography. Int. J. Adhes. Adhes..

[9] Taki K., Tomita B., Mizumachi H. (1982). Studies on aqueous vinyl polymer solution-isocyanate adhesives. Part I. Mechanical properties of base polymers and bond strength over a wide temperature range. Mokuzai Gakkaishi.

[10] Taki K., Tomita B., Mizumachi H. (1982). Studies on aqueous vinyl polymer solution-isocyanate adhesives. Part II. Dependence of mechanical properties and bond strength on the concentration of crosslinks in cured adhesives over a wide temperature range. Mokuzai Gakkaishi.

[11] Umemura K., Takahashi A., Kawai S. (1998). Durability of isocyanate resin adhesives for wood I: Thermal properties of isocyanate resin cured with water. J. Wood Sci..

[12] Guo J., Hu H., Zhang K., He Y., Guo X. (2018). Revealing the Mechanical Properties of Emulsion Polymer Isocyanate Film in Humid Environments. Polymers.

[13] Ling N., Hori N., Takemura A. (2008). Effect of postcure conditions on the dynamic mechanical behavior of water-based polymer-isocyanate adhesive for wood. J. Wood Sci..

[14] Zhang K., Hu H., Li S., He Y., Guo J. (2020). Effect of sodium dodecyl sulfate (SDS) on mechanical performance of polyvinyl-acetate-based emulsion polymer isocyanate. Int. J. Adhes. Adhes..

[15] Rindler A., Pöll C., Hansmann C., Müller U., Konnerth J. (2018). Moisture related elastic and viscoelastic behaviour of wood adhesives by means of in-situ nanoindentation. Int. J. Adhes. Adhes..

[16] Aicher S., Christian Z., Stapf G. (2015). Creep Testing of One-Component Polyurethane and Emulsion Polymer Isocyanate Adhesives for Structural Timber Bonding. For. Prod. J..

[17] Struik L.C.E. (1978). Physical Aging in Amorphous Polymers and Other Materials.

[18] Zhang X., Hu H., Guo M. (2015). Relaxation of a hydrophilic polymer induced by moisture desorption through the glass transition. Phys. Chem. Chem. Phys..

[19] Dorkenoo K.D., Pfromm P.H. (1999). Experimental evidence and theoretical analysis of physical aging in thin and thick amorphous glassy polymer films. J. Polym. Sci. Pol. Phys..

[20] Rittigstein P., Torkelson J.M. (2006). Polymer–nanoparticle interfacial interactions in polymer nanocomposites: Confinement effects on glass transition temperature and suppression of physical aging. J. Polym. Sci. Part B Polym. Phys..

[21] Cangialosi D., Boucher V., Alegria A., Colmenero J. (2012). Enhanced physical aging of polymer nanocomposites: The key role of the area to volume ratio. Polymer.

[22] Soma H., Nishitsuji S., Inoue T. (2012). Molecular weight dependence in a relaxation phenomenon at glassy state: Physical aging of polycarbonate. Polymer.

[23] Hu H., Fan X., He Y. (2019). A Coupled Thermodynamic Model for Transport Properties of Thin Films during Physical Aging. Polymers.

[24] Kim J., Koros W., Paul D.R. (2006). Effects of CO_2_ exposure and physical aging on the gas permeability of thin 6FDA-based polyimide membranes—Part 2. with crosslinking. J. Membr. Sci..

[25] Flory A., McKenna G.B. (2010). Physical aging behavior of the normal force and torque in polymer glasses. Mech. Time Depend. Mater..

[26] Hutchinson J.M. (1995). Physical aging of polymers. Prog. Polym. Sci..

[27] Cangialosi D., Boucher V., Alegria A., Colmenero J. (2013). Physical aging in polymers and polymer nanocomposites: Recent results and open questions. Soft Matter.

[28] Ling Z., Omura Y., Hori N., Iwata T., Takemura A. (2018). In-situ chemical structure analysis of aqueous vinyl polymer solution-isocyanate adhesive in post-cure process by using Fourier transform near infrared spectroscopy. Int. J. Adhes. Adhes..

[29] Kirkegaard P., Pederson N.J., Eldrup M. (1989). PATFIT-88: A Date-Processing System for Positron Annihilation Spectra on Mainframe and Personal Computers.

[30] Li X.S., Boyce M.C. (1993). On the measurement of structural relaxation in polymers using positron annihilation lifetime spectroscopy. J. Polym. Sci. Part B Polym. Phys..

[31] Cangialosi D., Schut H., Wübbenhorst M., Van Turnhout J., Van Veen A. (2003). Accumulation of charges in polycarbonate due to positron irradiation. Radiat. Phys. Chem..

[32] Curro J.G., Lagasse R.R., Simha R. (1982). Diffusion model for volume recovery in glasses. Macromolecules.

[33] Thornton A.W., Hill A.J., Nairn K.M., Hill J.M. (2008). Predicting particle transport through an aging polymer using vacancy diffusion. Curr. Appl. Phys..

[34] McCaig M., Paul D., Barlow J. (2000). Effect of film thickness on the changes in gas permeability of a glassy polyarylate due to physical agingPart II. Mathematical model. Polymer.

[35] Cangialosi D., Boucher V.M., Alegria A., Colmenero J. (2011). Free volume holes diffusion to describe physical aging in poly (mehtyl methacrylate)/silica nanocomposites. J. Chem. Phys..

[36] Doolittle A.K. (1951). Studies in Newtonian Flow. II. The Dependence of the viscosity of liquids on free-Space. J. Appl. Phys..

